# Supplementary material for: Exploiting natural chemical photosensitivity of anhydrotetracycline and tetracycline for dynamic and setpoint chemo-optogenetic control
Source: Nat Commun. 2020 Jul 31;11:3834. doi: 10.1038/s41467-020-17677-5 (PMC7395757; doi:10.1038/s41467-020-17677-5)
Supplement: Supplementary file 2 — Supplementary Information [file 41467_2020_17677_MOESM2_ESM.pdf]

Supplementary Information

for

Exploiting natural chemical photosensitivity of anhydrotetracycline and tetracycline for dynamic and setpoint chemo-optogenetic control

Baumschlager et al.

TetR - UVA exposure time raw data Figure 2a left

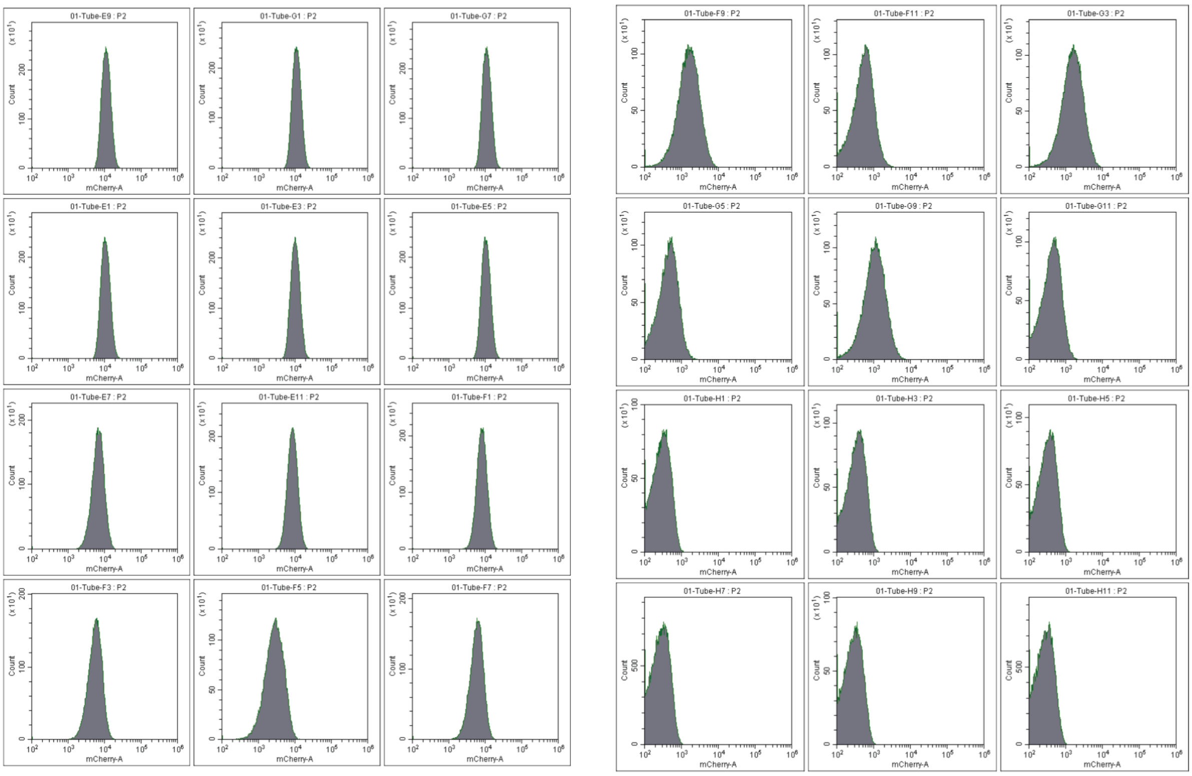

TetR - UVA intensity raw data Figure 2a right

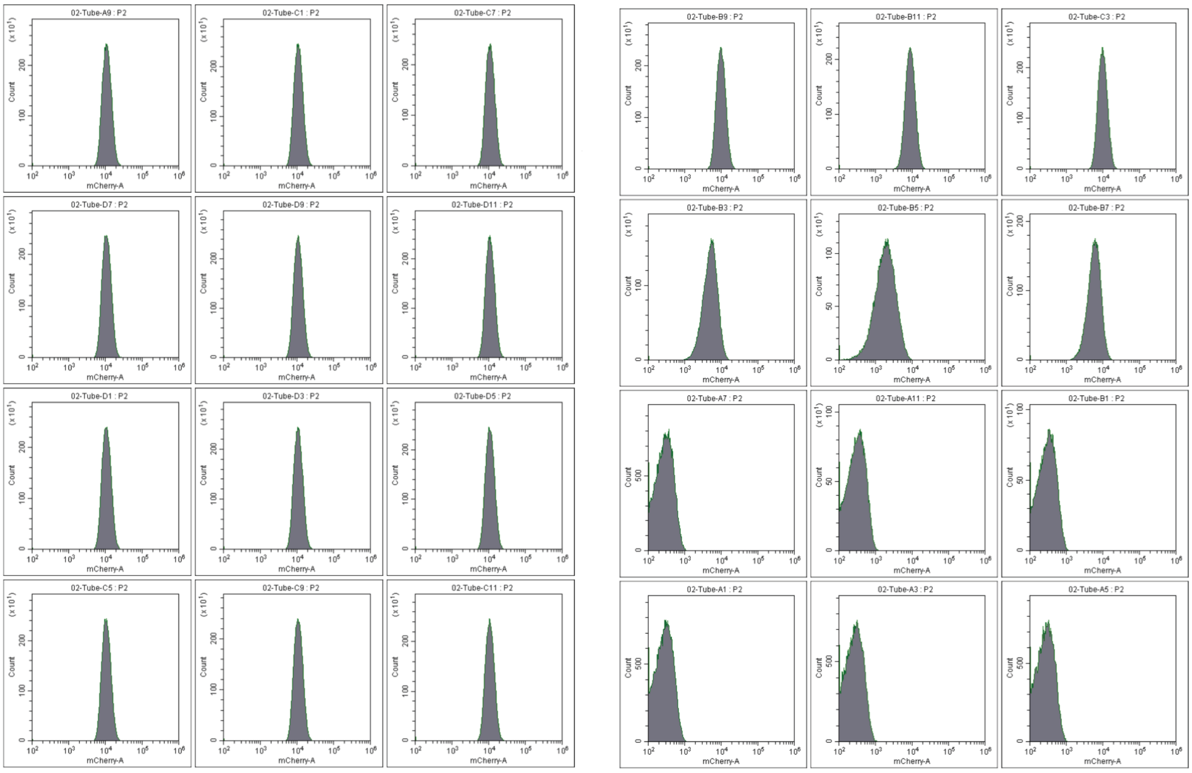

Supplementary Figure 1: Histograms of data shown in Fig.2a left for upper panel and Fig.2a right for lower panel. The triplicate measurements are shown next to each other and advance

with the left column downward, followed by the right column downward. The exposure time experiment (upper panel) shows cells incubated with 10 ng/ml aTc and 0, 30, 60, 90, 120, 150, 180 and 210 s with 375 nm light at intensity 4.1 mW/cm^2. The UVA intensity experiment (lower panel) shows cells incubated with 10 ng/ml aTc and 0, 0.064, 0.128, 0.256, 0.512, 1.028, 2.047 and 4.095 mW/cm^2 375nm light intensities and 4 min illumination time. The histograms show mCherry expression values of individual samples measured after 5h incubation.

rTetR - UVA exposure time raw data Figure 2b left

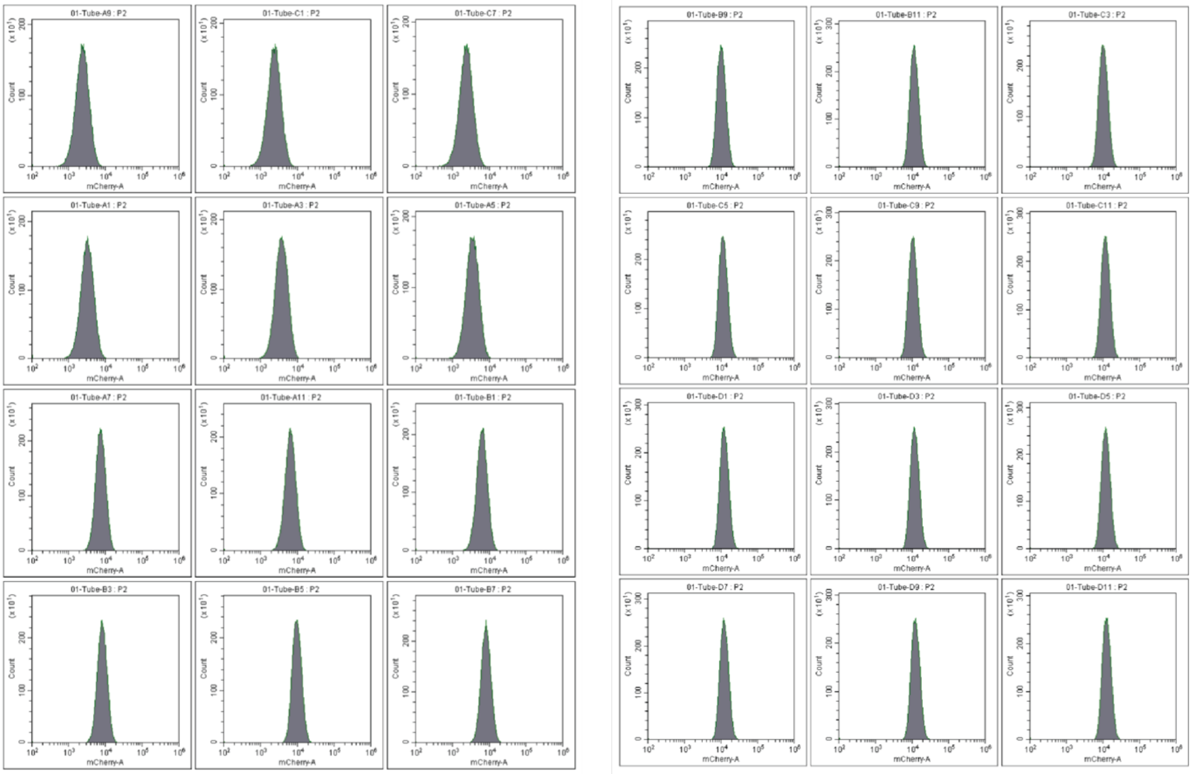

rTetR - UVA intensity raw data Figure 2b right

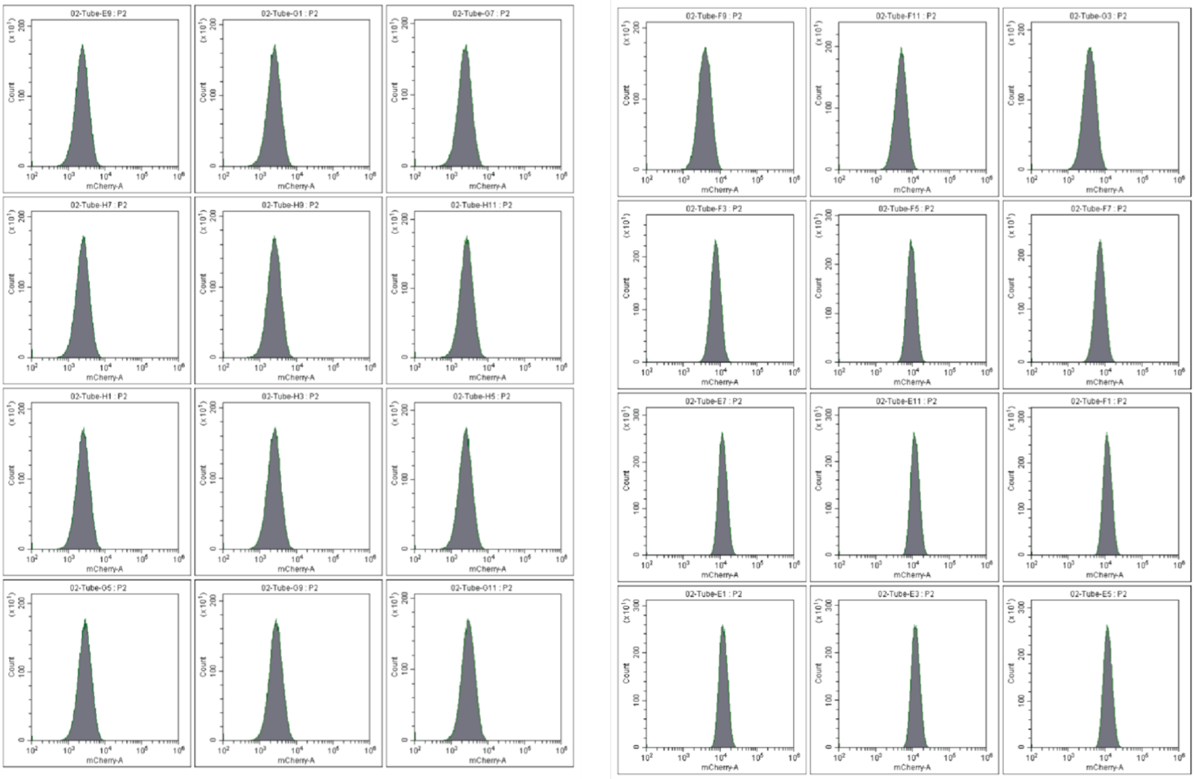

Supplementary Figure 2: Histograms of data shown in Fig.2b left for upper panel and Fig.2b right for lower panel. The triplicate measurements are shown next to each other and advance

with the left column downward, followed by the right column downward. The exposure time experiment (upper panel) shows cells incubated with 25 ng/ml aTc and 0, 30, 60, 90, 120, 150, 180 and 210 s with 375 nm light at intensity 4.1 mW/cm^2. The UVA intensity experiment (lower panel) shows cells incubated with 25 ng/ml aTc and 0, 0.064, 0.128, 0.256, 0.512, 1.028, 2.047 and 4.095 mW/cm^2 375nm light intensities and 4 min illumination time. The histograms show mCherry expression values of individual samples measured after 5h incubation.

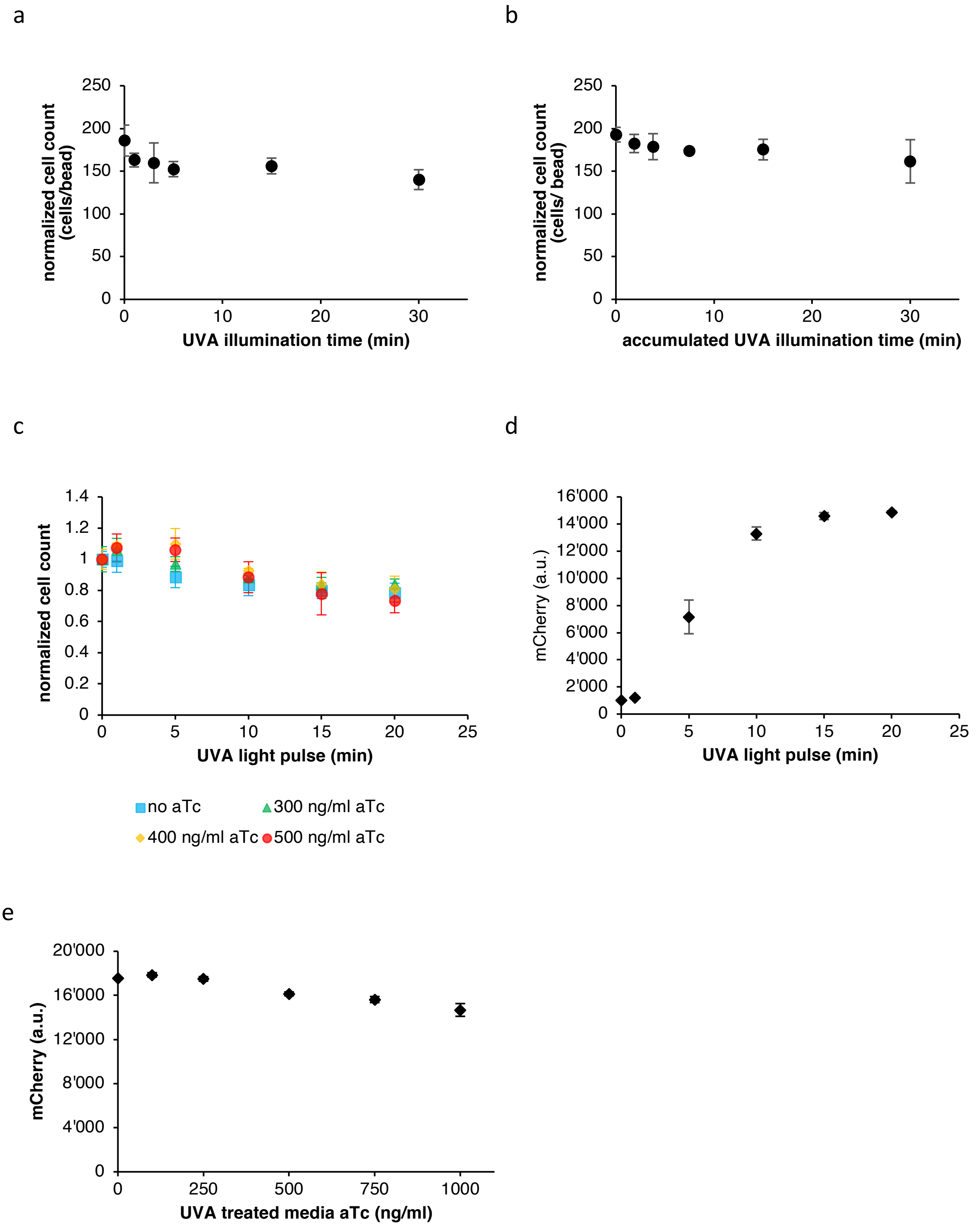

Supplementary Figure 3: Effect of UVA exposure and high aTc concentration inactivation on cell growth. Media was inoculated to an OD_{600} of about 0.00015, and the culture split to triplicates which were treated with different UVA exposure with the maximal intensity used

in this study and possible with our light setup (4.1 mW/cm^2). Differential cell growth was evaluated through cell counting. (a) A UVA light pulse of varying duration (x-axis) with the maximum intensity of our light setup is applied at the beginning of a 5h incubation period. (b) UVA is applied in pulses of maximal intensity throughout the experiment, which are shown as the sum of the applied duration on the x-axis. For both experiments shown in A and B, absolute cell counts are determined through counting beads as described in the methods section of the manuscript. Diagrams show mean values and standard deviation of three biological replicates of cell counts normalized to counting beads for growth experiments measured after 5 h incubation time in all cases. (c) Inactivation of 300, 400 and 500 ng/ml with increasing duration of UVA illumination at maximal intensity in comparison with cultures containing no aTc. The normalized cell count (cells/bead) was normalized to one for each of the individual samples to allow for comparison of cultures with slightly different initial inoculum. (d) mCherry expression level of 500 ng/ml aTc containing cultures incubated with increasing duration of UVA illumination at maximal intensity. (e) Inactivation of high aTc concentrations with UVA light. Media containing different concentrations of aTc was treated with a UVA pulse, before cells containing rTetR, which allows expression in the absence of aTc, were added. The final aTc concentrations after culture addition are shown on the x-axis. The diagrams show mean mCherry expression values and standard deviation of three biological replicates (n=3) measured after 5h incubation. Source data are provided as a Source Data file.

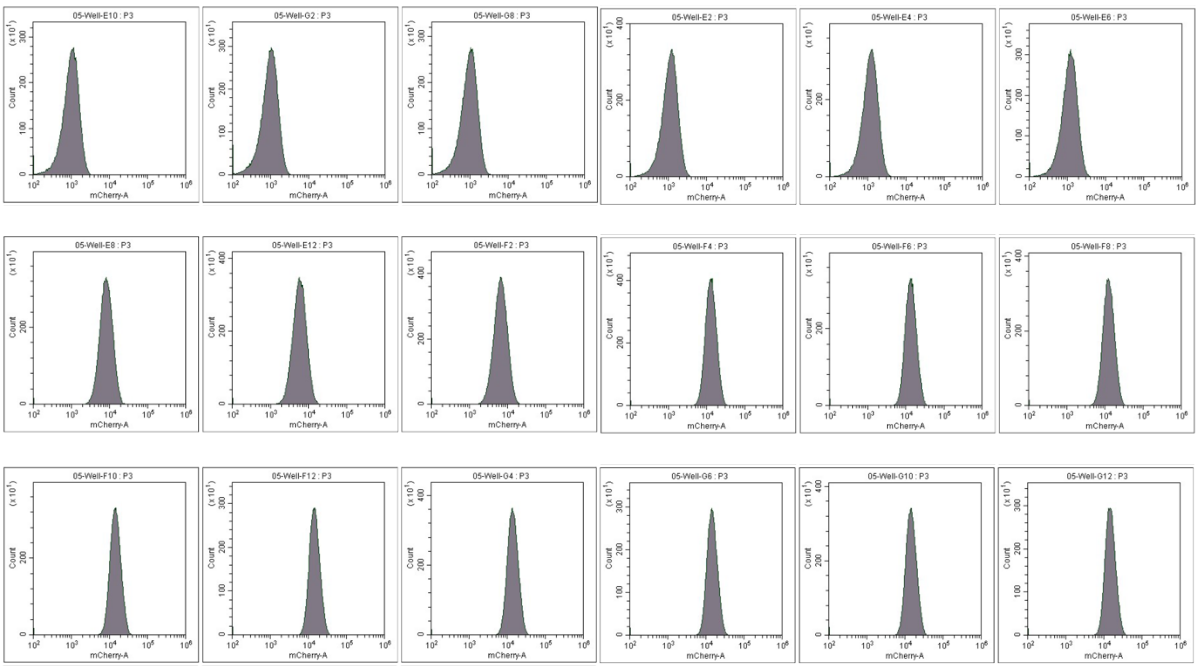

Supplementary Figure 4: Histograms of mCherry expression of triplicates with cells incubated with 500 ng/ml aTc (Fig.S3d) without UVA incubation (row 1, left 3 histograms) or incubated with UVA light pulses of 1 min (row 1, right 3 histograms), 5 min (row 2, left 3 histograms), 10 min (row 2, right 3 histograms), 15 min (row 3, left 3 histograms), and 20 min (row 3, right 3 histograms). The histograms show mCherry expression values of individual samples measured after 5h incubation.

a

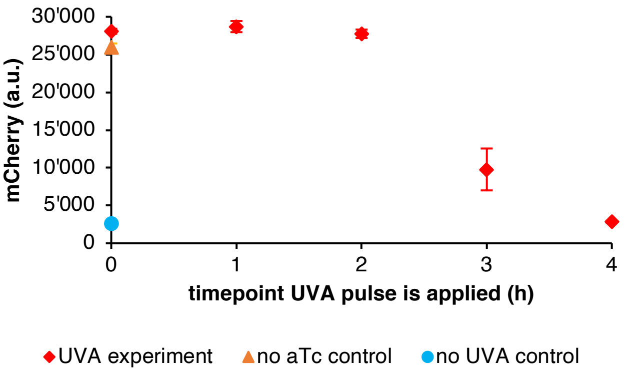

b

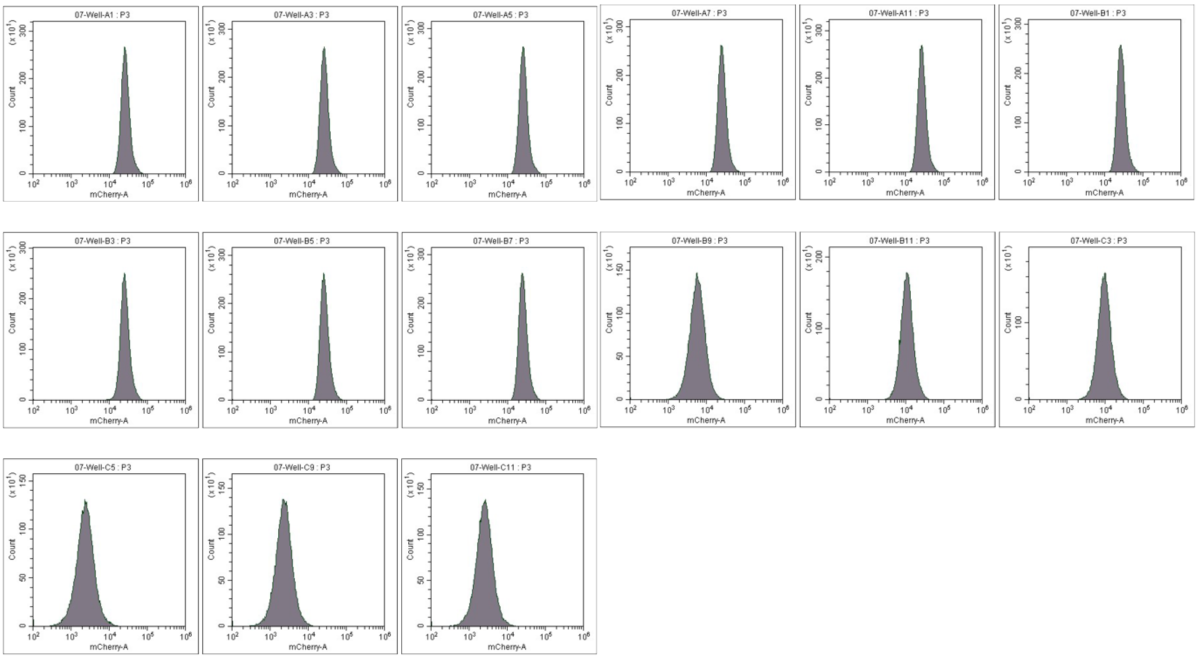

Supplementary Figure 5: (a) UVA inactivation at high cell densities. A pulse of UVA is applied to rTetR-containing cells at the start of incubation (timepoint 0) or after 1, 2, 3 and 4h of incubation for a total of 5h incubation time. We use our rTetR strain to visualize activation of gene expression *via* inactivation of aTc, as slower growth at higher cell density and resulting lower dilution rates does not allow for the detection of changes in aTc for the TetR strain. The culture was inoculated at an OD_{600} of 0.097 ± 0.002, aTc added to 25 ng/ml and the culture incubated as previously described for 5h at which the cultures reached an OD_{600} of 3.283 ± 0.006. We divided the culture at the beginning of the experiments and for triplicates, applied

a 15 min light pulse at the beginning, or applied the light with a delay of 1, 2, 3 and 4 hours after of incubation. Controls show the same culture without UVA (no UVA control) and without aTc addition (no aTc control). (b) Histograms of triplicates of the UVA experiment timepoints shown in Fig.2. UVA applied at the start (line 1, left 3 histograms), 1h (line 1, right 3 histograms), 2h (line 2, left 3 histograms), 3h (line 2, right 3 histograms), 4h (line 3 histograms) of the incubation. The diagram shows mean mCherry expression values and standard deviation of three biological replicates (n=3) measured after 5h incubation. The histograms show mCherry expression values of individual samples measured after 5h incubation. Source data are provided as a Source Data file.

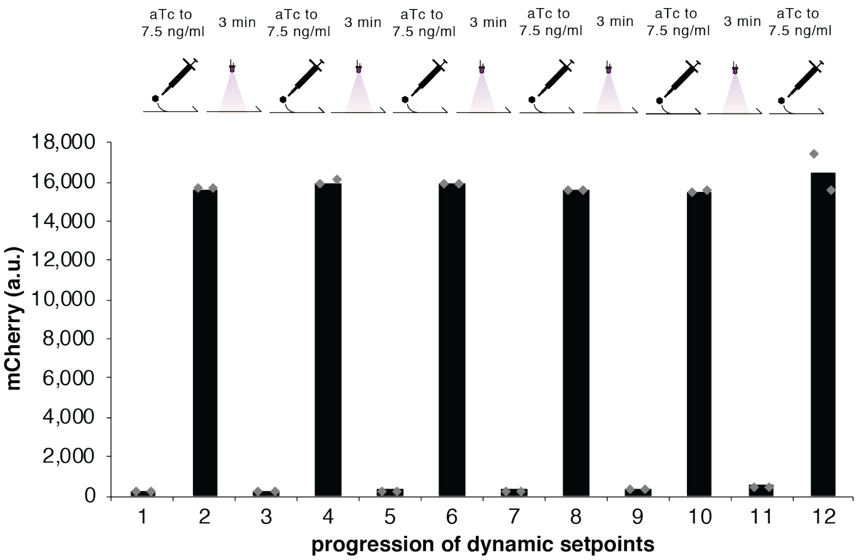

Supplementary Figure 6: On-off control using an "aTc toggle switch" through repeated addition and UVA inactivation of aTc using TetR controlled mCherry expression. The inputs were applied subsequently for each data point shown, as previously described for the dynamic protein expression experiment shown in Fig.3a. Starting from a culture without aTc in setpoint 1, aTc was alternately added to a concentration of 7.5 ng/ml, which already lies in the saturating expression regime for aTc, and removed via a 3 min UVA light pulse. The experiment was performed in duplicates (n=2; individual samples shown as grey diamonds) and the corresponding mean fluorescent value is depicted as black bars. Source data are provided as a Source Data file.

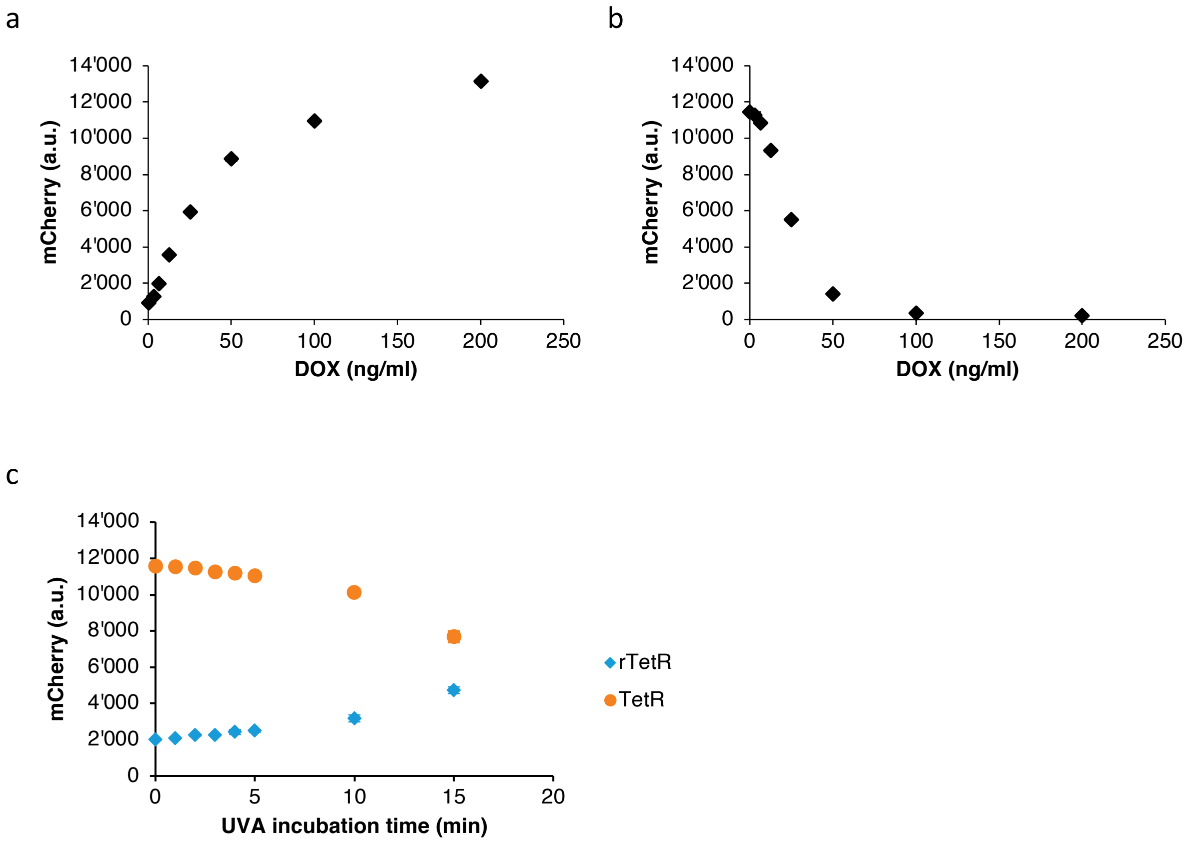

Supplementary Figure 7: UVA stability of doxycycline (DOX). (a) Dose-response curve of DOX concentration and mCherry expression for TetR-controlled gene expression. (b) Dose-response curve of aTc concentration and mCherry expression for rTetR-controlled gene expression. (c) DOX shows increased stability with UVA illumination compared to aTc and can be used if light stability is required. A non-saturating concentration of 50 ng/ml DOX was used for the TetR and the rTetR strain. The diagrams show mean mCherry expression values and standard deviation of three biological replicates (n=3) measured after 5h incubation. Source data are provided as a Source Data file.

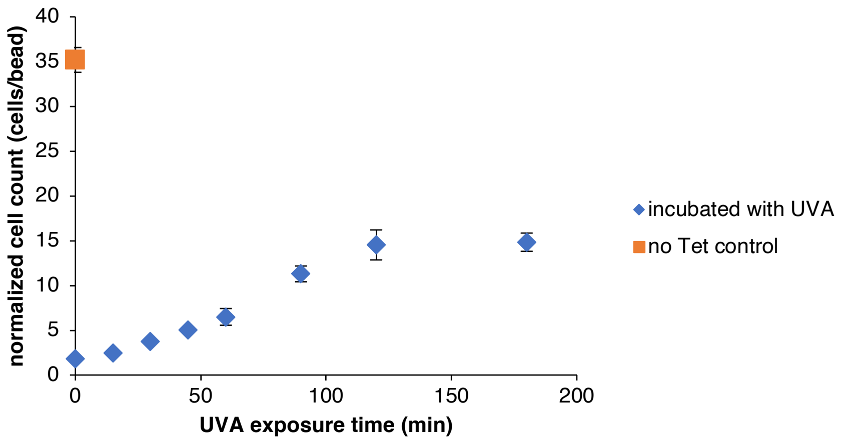

Supplementary Figure 8: Light-inducible control of growth rate with Tc in the presence of cells. The overnight culture was diluted 1:20,000 into fresh M9 medium, and then split into two samples, without Tc and with Tc at a final concentration of 400 ng/ml. The samples containing Tc were kept in the dark (t = 0) or exposed to UVA (4.1 mW/cm^2) with increasing illumination duration in a pseudo time course (blue diamonds). All samples were incubated for 5 h at 37°C after dilution of the overnight culture and addition of Tc. The control without Tc shows the highest cell counts, as the cells start growing immediately after dilution of the overnight culture, while the cells with 400 ng/ml Tc are inhibited. With increasing UVA exposure time, Tc is inactivated which leads to increasing cell counts. Tc is completely inactivated after 180 min (Fig.3d), however cannot reach the count value of the no Tc control as seen in Fig.3d due to the initial lag in growth. Diagrams show mean values and standard deviation of three biological replicates (n=3) of cell counts normalized to counting beads. Source data are provided as a Source Data file.

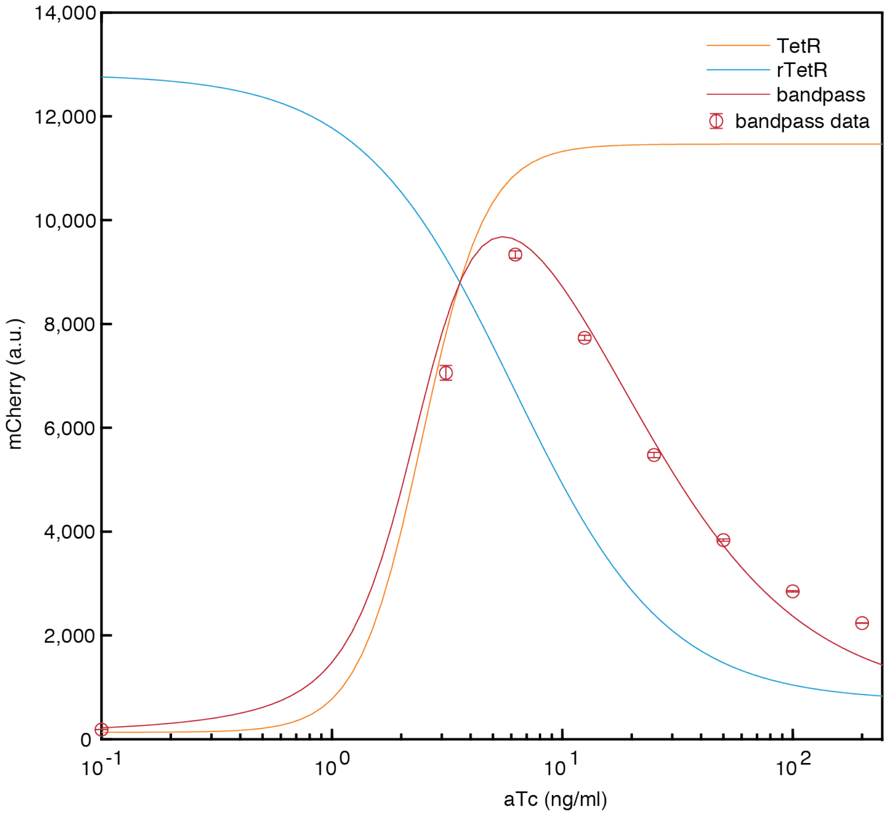

Supplementary Figure 9: Mathematical model of the aTc-dose response curve of the aTc bandpass filter shown in Fig.4a of the Manuscript. An empirical mathematical model of the bandpass filter (red line) was obtained through combining the dose response curves of TetR (orange line) and rTetR (blue line) and using the experimental data of the bandpass (red circles) to define the proportionality factor, which only required fitting of one parameter, while parameters obtained in previous fits of the dose-response curves were not modified as described in more detail in the Methods section. The diagram shows mean mCherry expression values and standard deviation of three biological replicates (n=3) measured after 5h incubation.

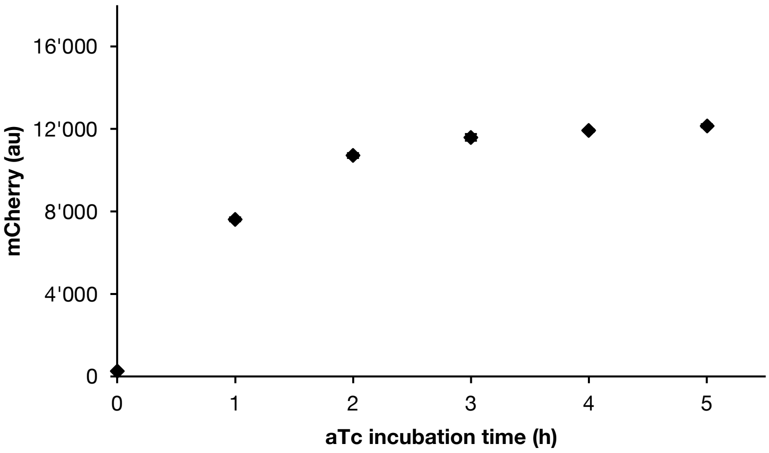

Supplementary Figure 10: Time course of aTc induction at logarithmic cell growth. Bacterial cells containing the TetR regulator controlling mCherry expression (Fig.1b) were diluted 1:20,000 from an overnight culture to an OD600 of ~ 0.0002 and incubated for 5h in 24-well plates. aTc was added to a concentration of 100 ng/ml at the start of the experiment, after 1, 2, 3, and 4h incubation and at the end of the experiment after 5h. This effectively creates a pseudo-time course. Steady state expression is reached after 3h and maintains constant. Cell cultures did not reach an OD600 of > 0.1 after 5h incubation, effectively maintaining logarithmic growth phase. Diagrams show mean values and standard deviation of at least two biological replicates (n=2). Source data are provided as a Source Data file.

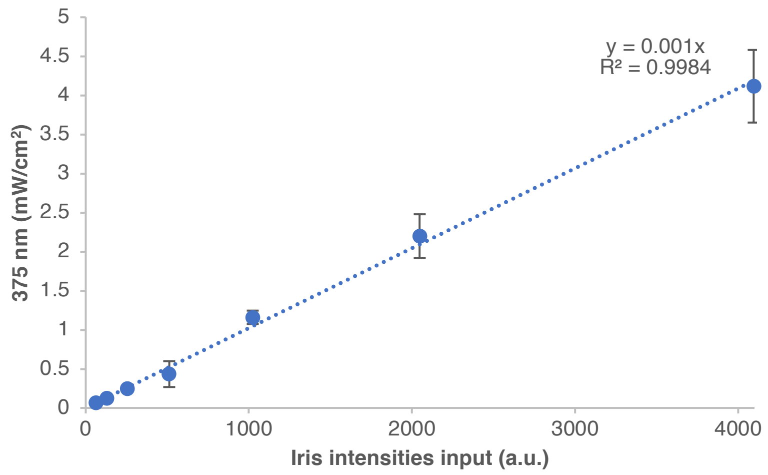

Supplementary Figure 11: UVA light intensity calibration of the LPA. Measured light intensities correspond linearly (equation in the diagram) to light inputs given through the Iris software after calibration of the device as described in the Methods section. The diagram shows mean measured light intensity values and standard deviation of three different LED positions (n=3). Source data are provided as a Source Data file.

Flow cytometry gating strategy for cell fluorescence measurements

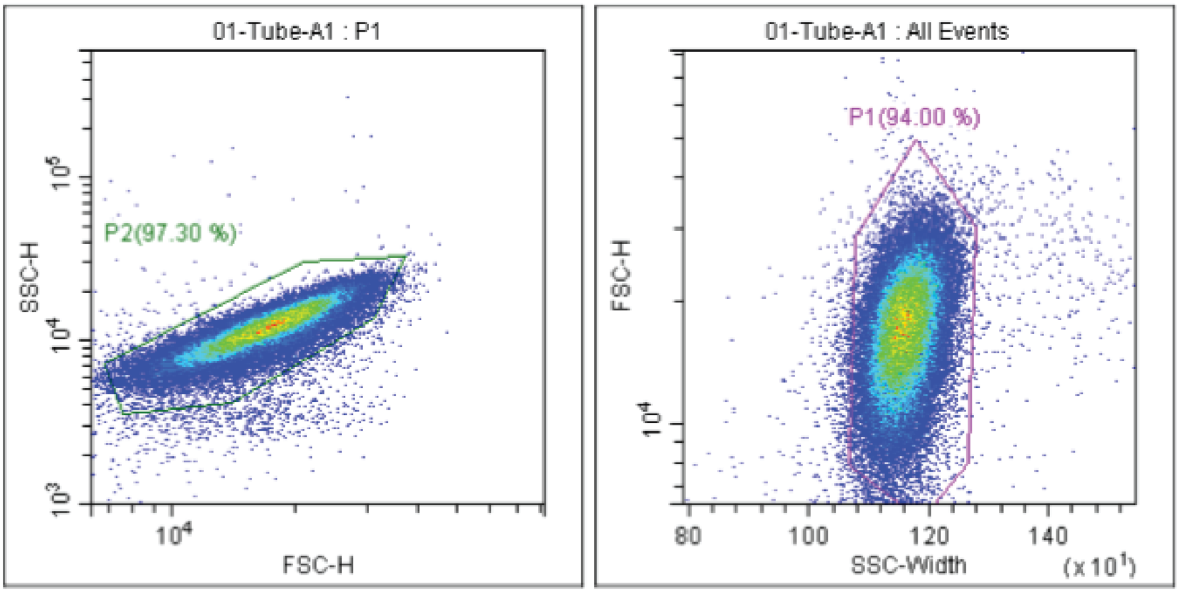

Flow cytometry gating strategy for cell growth measurements

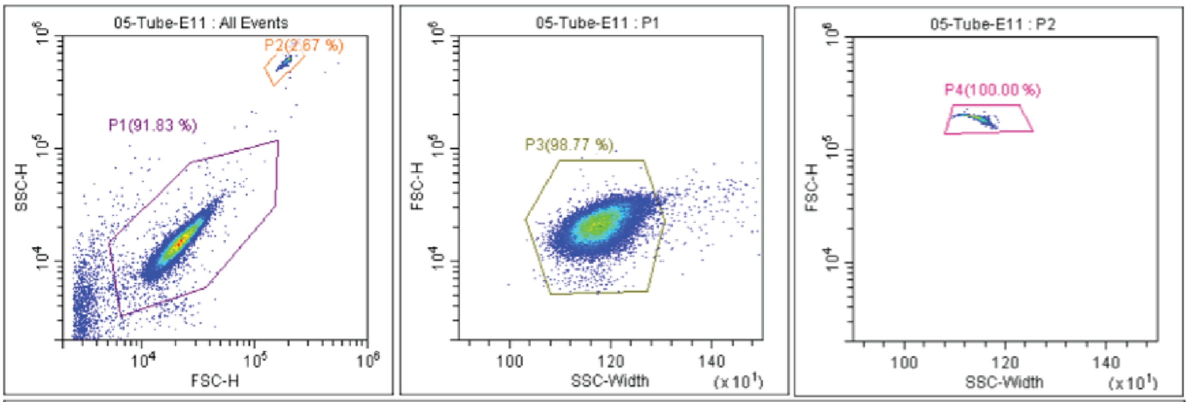

Tube Name: 05-Tube-E11
Sample ID:
Record Time: 2019-03-18 17:10:55

Population	Events
All Events	29824
P2	797
P1	27387
P3	27051
P4	797

Supplementary Figure 12: Flow cytometry gating strategies. For cell fluorescence measurements (upper panel), cells were first gated for singlets using the SSC-Width and FSC-H (gate P1, right panel) and then separated from other non-cellular particles using FSC-H and SSC-H (gate P2, left panel). For growth measurements (lower panel), determined as the ratio

of cells per defined volume of counting particles, FSC-H and SSC-H was used to separate cells (gate P1, left panel) and counting beads (gate P2, left panel) from other particles. Based on these gates, cells (gate P3, middle) and particles (gate P4, right) were again gated for singlets using SSC-Width and FSC-H. Shown are screenshots of gates are taken directly from the Cytoflex software. Gates were drawn by hand and kept constant for all experiments using the same cell type.

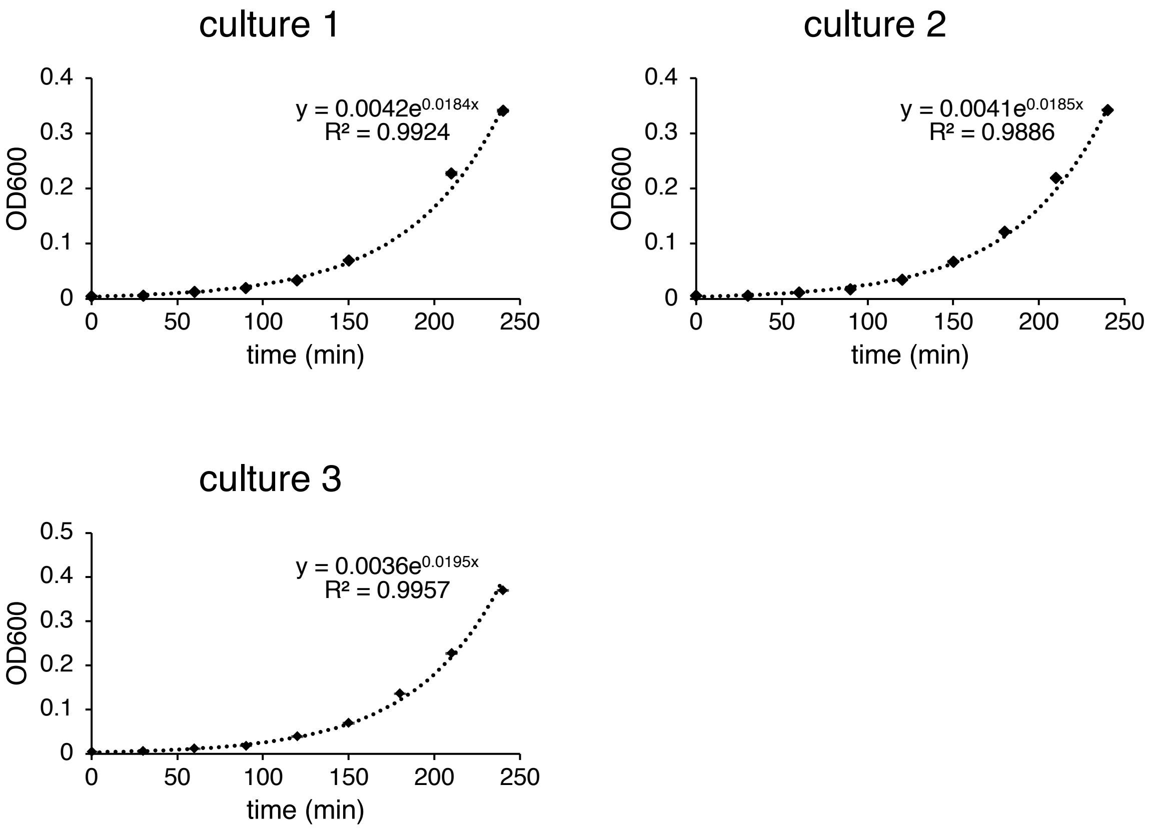

Supplementary Figure 13: Growth curves of *E.coli* strain AB859. Strain AB859 was used for UVA and aTc inactivation toxicity tests shown in Fig.S3. Shown are mean and standard deviation of technical replicates (n=3) for OD_{600} of cells grown in our 24-well setup as described in the Methods section. To obtain accurate measurements of the growth rate throughout this experiment, a starting OD above our detection limit was used (OD_{600} of 0.005). A Nanodrop^{TM} 2000c (Thermo Scientific) was used for all optical density measurements. Source data are provided as a Source Data file.

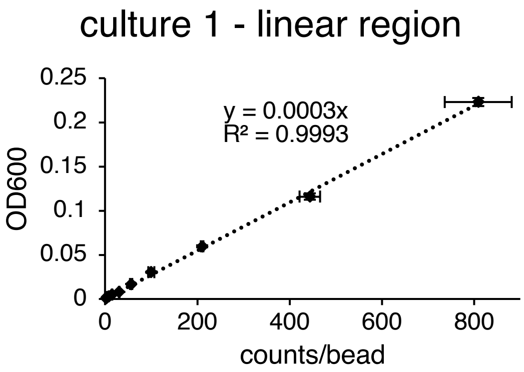

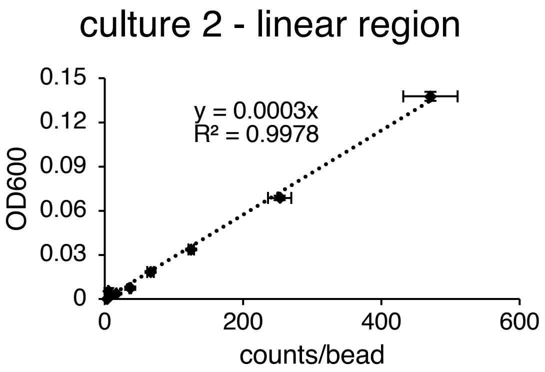

Supplementary Figure 14: Correlation of OD_{600} to counts/bead. Two independent cultures were inoculated from overnight cultures to an optical density of 0.1 and grown until they reached an optical density of about 0.5 to transition the cells into log growth phase. The cultures were then serially diluted and then the OD_{600} measured in technical triplicates, as well as three samples added to the inhibition solution containing counting beads (Methods section) for each dilution. Shown are mean and standard deviation of technical replicates (n=3) for OD_{600} and separated pipetted replicates for cell counting. Source data are provided as a Source Data file.

Supplementary Table 1: Parameter values obtained through fitting of Hill- and Hill-like equation as well as the exponential decay equations shown in section “Mathematical modelling”

**Parameter**	**Value**
c (light intensity)	1.5206
c (light duration)	0.0178
a (atc)	134
R_{max} (atc)	11331
K_m (atc)	16.7
n (atc)	3.126
a (ratc)	12815
R_{max} (ratc)	734
K_m (ratc)	10.6
n (ratc)	1.303
