# Supplementary material for: Exploiting natural chemical photosensitivity of anhydrotetracycline and tetracycline for dynamic and setpoint chemo-optogenetic control
Source: Nat Commun. 2020 Jul 31;11:3834. doi: 10.1038/s41467-020-17677-5 (PMC7395757; doi:10.1038/s41467-020-17677-5)
Supplement: Supplementary file 3 — Reporting Summary [file 41467_2020_17677_MOESM3_ESM.pdf]

nature research

Corresponding author(s): Mustafa Khammash

Last updated by author(s): Jun 22, 2020

Reporting Summary

Nature Research wishes to improve the reproducibility of the work that we publish. This form provides structure for consistency and transparency in reporting. For further information on Nature Research policies, see Authors & Referees and the Editorial Policy Checklist .

Statistics

For all statistical analyses, confirm that the following items are present in the figure legend, table legend, main text, or Methods section.

n/a	Confirmed	
[ ]	[x]	The exact sample size (*n*) for each experimental group/condition, given as a discrete number and unit of measurement
[ ]	[x]	A statement on whether measurements were taken from distinct samples or whether the same sample was measured repeatedly
[x]	[ ]	The statistical test(s) used AND whether they are one- or two-sided<br>*Only common tests should be described solely by name; describe more complex techniques in the Methods section.*
[x]	[ ]	A description of all covariates tested
[x]	[ ]	A description of any assumptions or corrections, such as tests of normality and adjustment for multiple comparisons
[ ]	[x]	A full description of the statistical parameters including central tendency (e.g. means) or other basic estimates (e.g. regression coefficient) AND variation (e.g. standard deviation) or associated estimates of uncertainty (e.g. confidence intervals)
[x]	[ ]	For null hypothesis testing, the test statistic (e.g. *F*, *t*, *r*) with confidence intervals, effect sizes, degrees of freedom and *P* value noted<br>*Give P values as exact values whenever suitable.*
[x]	[ ]	For Bayesian analysis, information on the choice of priors and Markov chain Monte Carlo settings
[x]	[ ]	For hierarchical and complex designs, identification of the appropriate level for tests and full reporting of outcomes
[x]	[ ]	Estimates of effect sizes (e.g. Cohen's *d*, Pearson's *r*), indicating how they were calculated

Our web collection on statistics for biologists contains articles on many of the points above.

Software and code

Policy information about availability of computer code

Data collection	CytExpert 2.1.092 (Beckman Coulter)
Data analysis	CytExpert 2.1.092 (Beckman Coulter), Microsoft Excel for Mac Version 16.16.9, Matlab R2015a 8.5.0.197613 (Mathworks)

For manuscripts utilizing custom algorithms or software that are central to the research but not yet described in published literature, software must be made available to editors/reviewers. We strongly encourage code deposition in a community repository (e.g. GitHub). See the Nature Research guidelines for submitting code & software for further information.

Data

Policy information about availability of data

All manuscripts must include a data availability statement. This statement should provide the following information, where applicable:

- Accession codes, unique identifiers, or web links for publicly available datasets
- A list of figures that have associated raw data
- A description of any restrictions on data availability

The datasets generated during this study are available in the 'Source Data' file as well as from the corresponding author upon reasonable request.

Field-specific reporting

Please select the one below that is the best fit for your research. If you are not sure, read the appropriate sections before making your selection.

[x] Life sciences [ ] Behavioural & social sciences [ ] Ecological, evolutionary & environmental sciences

For a reference copy of the document with all sections, see nature.com/documents/nr-reporting-summary-flat.pdf

October 2018

Life sciences study design

All studies must disclose on these points even when the disclosure is negative.

Sample size	Each experiment in the main text was performed in triplicates and is on par with standard bacterial experiments reported in the field.
Data exclusions	No data were excluded from analysis.
Replication	A minimum of three biological replicates for main text figures were used and are indicated in the figure legends. All findings were reliably reproducible.
Randomization	Bacterial cultures were propagated from isogenic stocks derived from randomly selected colonies. When splitting cultures into different conditions as well as sampling cultures for measurements, cells within the cultures are randomly sampled as there is no control over which cells are selected.
Blinding	Researchers were not blinded during experiments because the experiments were not based on qualitative scoring metrics. In addition, each bacterial culture measurement is a random sampling of cells within a culture with no selection bias.

Reporting for specific materials, systems and methods

We require information from authors about some types of materials, experimental systems and methods used in many studies. Here, indicate whether each material, system or method listed is relevant to your study. If you are not sure if a list item applies to your research, read the appropriate section before selecting a response.

Materials & experimental systems

n/a	Involved in the study	
☒	☐	Antibodies
☒	☐	Eukaryotic cell lines
☒	☐	Palaeontology
☒	☐	Animals and other organisms
☒	☐	Human research participants
☒	☐	Clinical data

Methods

n/a	Involved in the study	
☒	☐	ChIP-seq
☐	☒	Flow cytometry
☒	☐	MRI-based neuroimaging

Flow Cytometry

Plots

Confirm that:

- ☒ The axis labels state the marker and fluorochrome used (e.g. CD4-FITC).
- ☒ The axis scales are clearly visible. Include numbers along axes only for bottom left plot of group (a 'group' is an analysis of identical markers).
- ☒ All plots are contour plots with outliers or pseudocolor plots.
- ☒ A numerical value for number of cells or percentage (with statistics) is provided.

Methodology

Sample preparation	Fluorescence measurements: Cells grown in minimal media were sampled and mixed with the same volume of 500 µg/ ml rifampicin and 50 µg/ ml tetracycline in phosphate-buffered saline. mCherry fluorescence was matured for 90 min at 37°C and read undiluted on the flow cytometer.<br>Cell count measurements: 100 µl samples of cells grown in minimal media were mixed with 79 µl of 500 µg/ ml rifampicin and 50 µg/ml tetracycline in phosphate-buffered saline and 21 µl of 2 µm AccuCount Blank Particles (Spherotech) before being measured on a flow cytometer.
Instrument	a Cytoflex S flow cytometer (Beckman Coulter) equipped with CytExpert 2.1.092 software.
Software	CytExpert 2.1.092 (Beckman Coulter)
Cell population abundance	No cell sorting was performed.
Gating strategy	mCherry fluorescence was measured with a 561 nm laser and 610/20 nm band pass filter and following gain settings: forward scatter 100, side scatter 100, mCherry gain 1,500 when mCherry was expressed from aTc-regulated promoters and 300 gain when mCherry was expressed with Opto-T7RNAP due to the difference in expression levels. Thresholds of 2,500 FSC-H and 1,000 SSC-H were used for all samples. The flow cytometer was calibrated before each experiment with QC beads (CytoFLEX Daily QC Fluorospheres, Beckman Coulter) to ensure comparable fluorescence values across experiments from different days. At least

15,000 events were recorded in a two-dimensional forward and side scatter gate, which was drawn by eye and corresponded to the experimentally determined size of the testing strain at logarithmic growth and was kept constant for analysis of all experiments and used for calculations of the fluorescence mean using the CytExpert software.

☒ Tick this box to confirm that a figure exemplifying the gating strategy is provided in the Supplementary Information.
