# Supplementary material for: Exploiting natural chemical photosensitivity of anhydrotetracycline and tetracycline for dynamic and setpoint chemo-optogenetic control
Source: Nat Commun. 2020 Jul 31;11:3834. doi: 10.1038/s41467-020-17677-5 (PMC7395757; doi:10.1038/s41467-020-17677-5)
Supplement: Supplementary file 4 — Description of Additional Supplementary Files [file 41467_2020_17677_MOESM4_ESM.pdf]

Title: Supplementary Data 1

Description: The Supplementary Data 1 file contains novel plasmids and genetic parts used in this study.
